# The Mandrillus Face Database: A portrait image database for individual and sex recognition, and age prediction in a non-human primate

**DOI:** 10.1016/j.dib.2023.108939

**Published:** 2023-01-25

**Authors:** Sonia Tieo, Claudia Ximena Restrepo-Ortiz, Berta Roura-Torres, Loic Sauvadet, Mélanie Harté, Marie J.E. Charpentier, Julien P. Renoult

**Affiliations:** a CEFE, University of Montpellier, CNRS, EPHE, IRD, Montpellier, France; b MARBEC, University of Montpellier, CNRS, Ifremer, IRD, Montpellier, France; c Behavioural Ecology & Sociobiology Unit, German Primate Center, Leibniz Institute of Primate Research, Kellnerweg 4, 37077 Gottingen, Germany; d Department of Sociobiology/Anthropology, Johann-Friedrich-Blumenbach, Institute of Zoology and Anthropology, Georg-August University Gottingen, Kellnerweg 6, 37077 Gottingen, Germany; e Projet Mandrillus, Fondation Lékédi Biodiversité, Bakoumba, Gabon; f Institut des Sciences de l'Evolution de Montpellier (ISEM), UMR5554 - University of Montpellier/CNRS/IRD/EPHE, Place Eugène Bataillon, 34095 Montpellier Cedex 5, France; g Department for the Ecology of Animal Societies, Max Planck Institute of Animal Behavior, Bücklestraβe 5, Konstanz 78467, Germany

**Keywords:** Face recognition, Artificial intelligence, Deep learning, *Mandrillus sphinx*, Primate

## Abstract

The Mandrillus Project is a long-term field research project in ecology and evolutionary biology, monitoring, since 2012, a natural population of mandrills (*Mandrillus sphinx;* primate) located in Southern Gabon. The Mandrillus Face Database was launched at the beginning of the project and now contains 29,495 photographic portraits collected on 397 individuals from this population, from birth to death for some of them. Portrait images have been obtained by manually processing images taken in the field with DSLR cameras: faces have been cropped to remove the ears and rotated to align the eyes horizontally. The database provides portrait images resized to 224 × 224 pixels associated with several manually annotated labels: individual identity, sex, age, face view, and image quality. Labels are stored within the image metadata and in a table accompanying the image database. This database will allow training and comparing methods on individual and sex recognition, and age prediction in a non-human animal.

**Specifications Table**

**Table:** 

Subject	Ecology
Specific subject area	Ecology and animal behaviour.
Type of data	JPG Images and Table of Metadata (csv file)
How the data were acquired	The photographic portraits were acquired on wild mandrills daily monitored in their natural environment, using different models of digital SLR cameras equipped with a long-focal lens (varying from 70 to 500 mm depending on camera models and distance to the subjects).
Data format	Raw (JPG)
Description of data collection	The Mandrillus Face Database contains 29,495 portrait photos of wild mandrills taken between 2012 and 2021, stored in jpg format with a 224 × 224 pixel resolution. These individuals are daily monitored by field assistants, who take pictures and record individuals’ identity directly in the field. Pictures are taken on both sexes of all ages (infants, juveniles, adolescents and adults). Data were normalized by manually cropping faces and rotating images to align the eyes along a horizontal line.
Data source location	· Institution: Centre d'Ecologie Fonctionnelle et Evolutive (CEFE), UMR5175, University of Montpellier/CNRS/IRD/EPHE, Montpellier, France Institut des Sciences de l'Evolution de Montpellier (ISEM), UMR5554, University of Montpellier/CNRS/IRD/EPHE, Montpellier, France · City/Town/Region: Lékédi Park, Bakoumba (GPS coordinates: -1.7974667244585478, 13.020375049984194 - accuracy:10 km) Country: Gabon
Data accessibility	· Repository name: Mandrillus Face Database Data identification number: 10.5281/zenodo.7467318 · Direct URL to data: https://doi.org/10.5281/zenodo.7467318

## Value of the Data

• This is the first public database that contains annotated face pictures of wild mandrills living in their natural environment. To our knowledge, this is also the largest photographic portrait database for non-human animals in the wild regarding the number of sampled individuals and the time frame (397 individuals totaling 29,495 pictures taken during 10 years)
• Pictures are labelled by experts in primatology for applications in Computer Vision, Machine Learning/Deep Learning, Data Science.
• The database is specifically designed to benchmark methods of individual recognition, face verification, sex recognition, and age prediction in a non-human primate

## 1 Objective

This database is used to study the role of face attributes in visual communication in wild animals. Beyond applications in behavioral ecology, the database is also currently used to develop and compare Deep Learning methods of individual recognition, face verification, sex recognition, and age prediction in a non-human primate. The database also allows training Deep Learning models to automatically pre-process future data (automatic cropping, alignment, and labelling of face view and image quality).

## 2 Data description

### 2.1 “Images” Folder

**Fig. 1:**
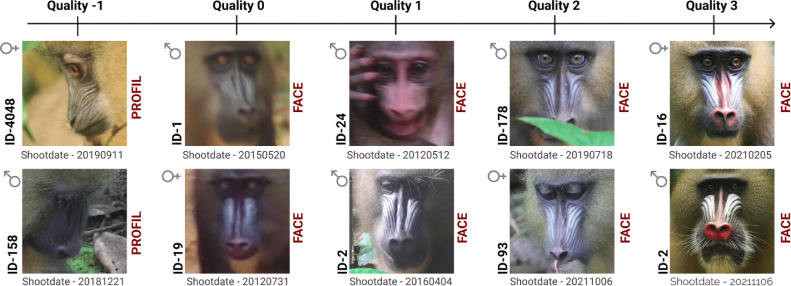
Sample of pictures collected on 10 individuals of both sexes and different ages, at different dates of shooting and of different quality and face view (see explanation of keywords below).

The core dataset of the Mandrillus Face Database are photographic portraits in jpg format with a 224 × 224 pixel resolution, obtained following a processing step. These portraits are stored in a folder named “Images”. The folder contains subfolders, each subfolder including all images from one individual taken across the course of the study. Subfolders are named after individual identifiers. The image name follows the same syntax: date of shooting (YYYYMMDD) followed by ‘id’ and the individual identifier, and eventually the numbering of the pictures (whenever several pictures of the same individual were taken on the same day), with these three parts separated by an underscore. For instance, “20190313_id170_4.jpg” represents the 4^th^ picture taken on an individual named 170 the 13^th^ of March 2019. Each picture is annotated with labels stored in the XMP and IPTC metadata of the photo itself (see details of the labels below). Metadata includes information related to the individual (individual-specific metadata; e.g. identifier, sex, date of birth) or to the image (image-specific metadata; e.g. face view and image quality). See Fig. 1 for a sample of images of different qualities.

### 2.2 MFD_metadata.csv file

This metadata file is a .csv file with 29,496 rows (first row contains the name of each column; and one row per picture) and 9 columns (attributes of each picture). Columns, arranged in that order in the file, contain the following information:

Photo_Name (type ‘string’): this column indicates the name of the picture (see above for the syntax). If the date of shooting is unknown, the date is “unknown” instead of “YYYYMMDD” format.
Id (type ‘integer’): this column provides the identifier of the individual depicted in the picture.
Sex (type ‘categorical’): this column indicates the sex of the individual on the picture (“f” for female, “m” for male and “unknown” if the sex was unknown).
dob (type ‘date’): this column gives the date of birth of the individual on the picture (with “YYYYMMDD” format). If unknown, this cell returns “NaN”.
dob_estimated (type ‘boolean’): this column indicates whether the date of birth is known with certainty (“False”) or whether it has been estimated by the field assistants, based on observational data on the mother's ovulation cycle (“True”). If the date of birth is unknown, this cell returns “NaN”.
error_dob (type ‘integer’): if ‘dob_estimated = ‘True’, this column indicates the uncertainty (measured in days) around the date of birth. If ‘dob_estimated = ‘False’, this cell returns 0. If the dob is unknown, this cell returns “NaN”.

FaceView (type ‘integer’): this column indicates whether the mandrill's face depicted on the picture is in frontal (1) or in profile (0) view. The face is considered to be frontal when both eyes are visible and the face is fully frontal or on 3/4 (approximately <30°) and the occlusion covers less than 50% of the face, otherwise the face is considered to be in profile view. The database includes 26,846 frontal and 2,649 profile pictures.

FaceQual (type ‘categorical): this column indicates the quality of the picture, ranging from 0 to 3 (or -1: when the quality has not been evaluated because the individual is in profile view). 0: pictures of bad quality and for which experienced field assistants are unable to recognize the individual from the picture alone, without the contextual information. 1: pictures of average quality for which experienced field assistants are able to recognize the individual from the picture alone, with some difficulties but without any contextual information. 2: pictures of good quality for which individual recognition is straightforward but the portrait does not meet the criteria of quality 3. 3: pictures of high quality for which individual recognition is straightforward, and the face has a neutral expression and is in perfect frontal view, with no shadow, bright spot or partial occlusion (“id card-like” portraits). The majority of the portraits are of quality 2 and 3 (see Fig. 4).

Shootdate (type ‘date’): date of shooting (“YYYYMMDD”).

**Fig. 2:**
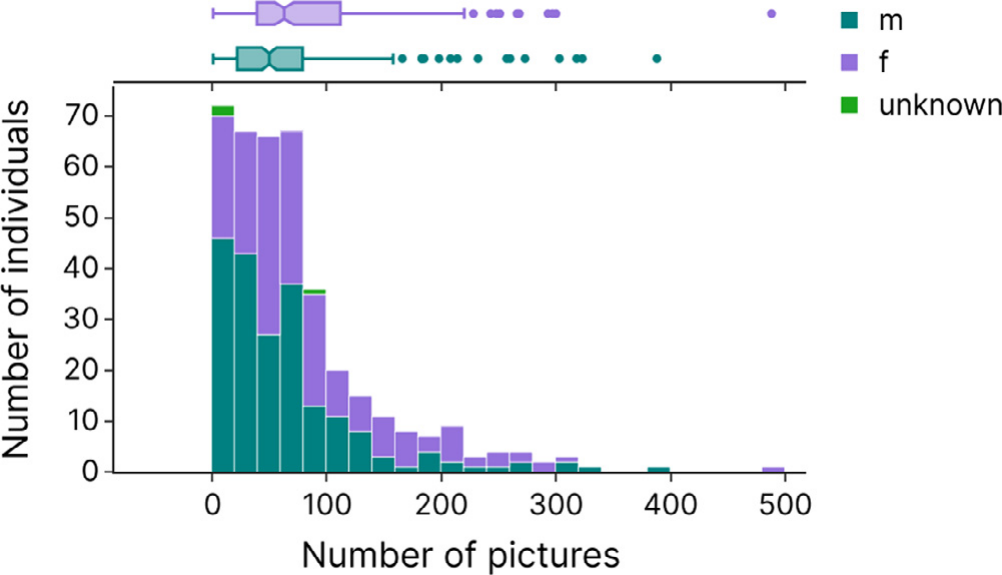
Histogram of the number of individuals (coloured by sex) as a function of the number of pictures. The bin size represents 20 pictures. Marginal boxplots at the top display the minimum (e.g. 1 picture in females and males), first quartile (e.g. 39 pictures in females and 22 in males), median (e.g. 63 pictures in females and 50 in males), third quartile (e.g. 112 pictures in females and 79 pictures in males), and maximum (e.g. 488 in females and 388 in males) pictures for females (purple) and males (green).

The database includes 191 females, 203 males and 3 individuals of unknown sex (infants aged less than a year, only). Fig. 2 represents the number of males and females as a function of the number of pictures collected on them, and the number of pictures per sex. On average, females are represented by 85±72 pictures and males by 65±64 pictures (here average are distorted by extreme values: as the boxplot shows, there are several individuals represented by only one picture, and one individual with 488 pictures). Fig. 2 also illustrates that the database contains numerous individuals with a few pictures, although 323 individuals are represented by more than 20 pictures.

**Fig. 3:**
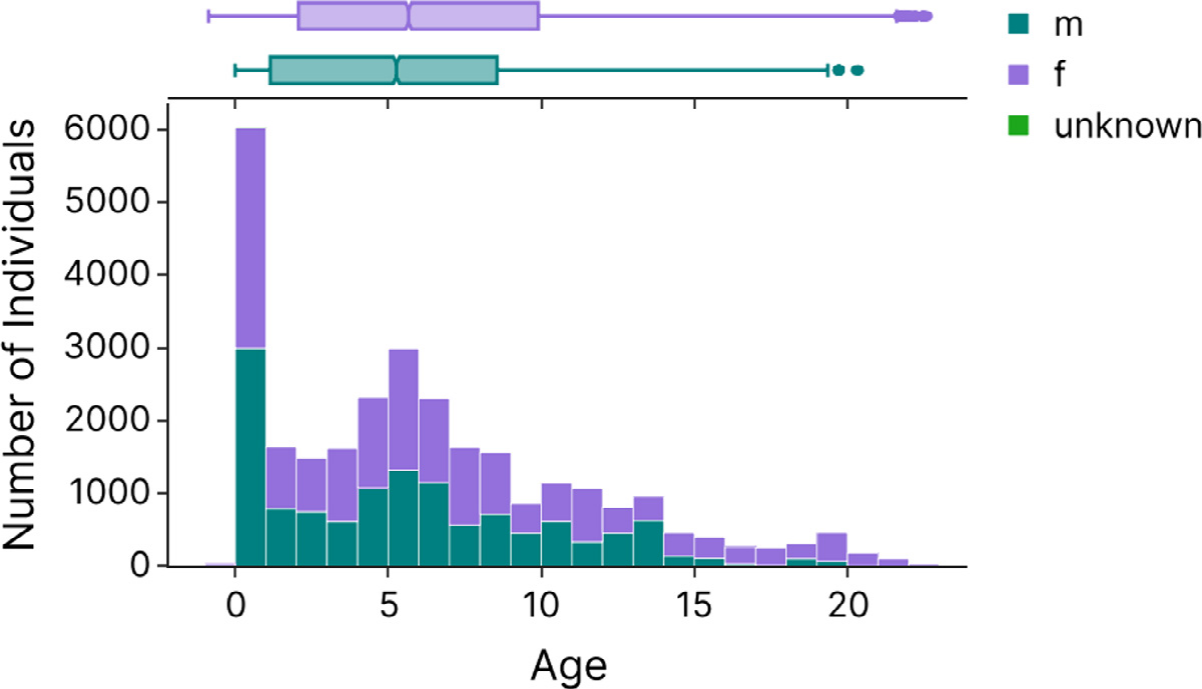
Histogram of the number of individuals (coloured by sex) as a function of their age. The bin size is 1 year old. Marginal boxplots of the ages at the top display the minimum (e.g. <1 year old in females and males), first quartile, median (e.g. 5.66 years old in females and 5.26 years old in males), third quartile, and maximum (e.g. 22.6 years old in females and 20 years old in males) ages for females (purple) and males (green).

The database contains individuals from birth to 23 years old. The age is calculated as the difference between the shooting date (“Shootdate” column) and the date of birth (“dob” column). Fig. 3 provides the histogram of the age distribution of the portrayed individuals with infants (0-1 year) corresponding to 20% of the total number of pictures.

**Fig. 4:**
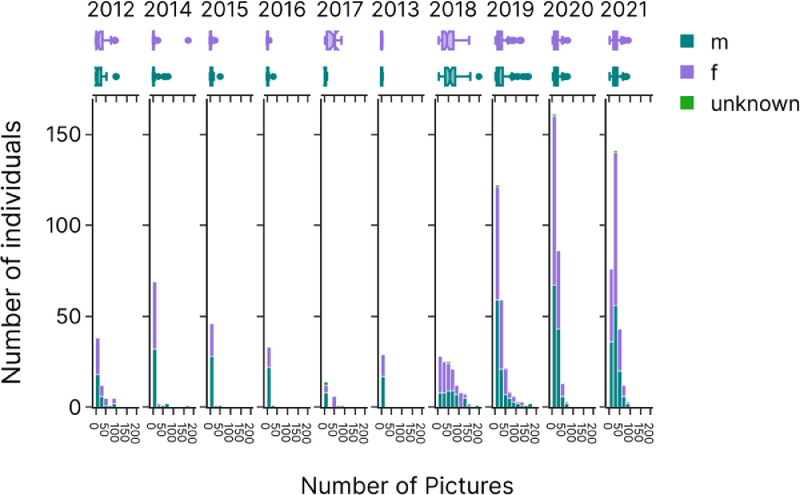
Yearly histograms of the number of individuals (coloured by sex) as a function of the number of pictures taken. The bin size represents 20 pictures. Marginal boxplots at the top display the minimum, first quartile, median, third quartile and maximum pictures for females (purple) and males (green), each year.

The Fig. 4 represents the number of pictures collected per year (extracted from the “ShootDate” column). Most pictures were taken from 2018 onward (more than 87% of the pictures).

**Fig. 5:**
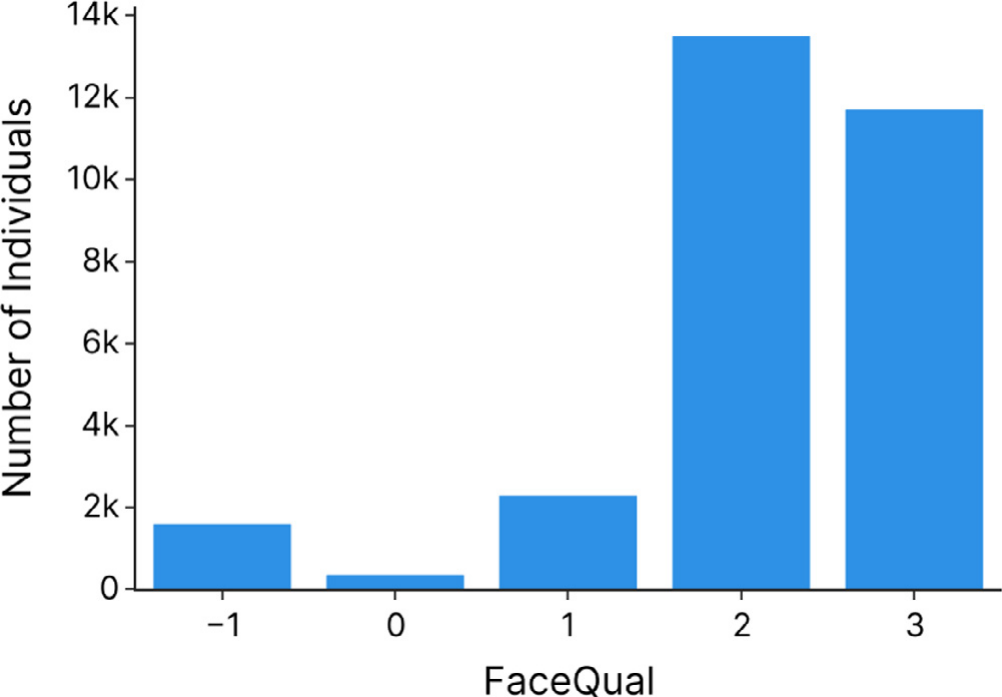
Barplot of the number of pictures for each quality score.

Finally, Fig. 5 represents the number of photos per quality score (“FaceQual” column). Most pictures (85%) are of quality 3 and 4.

## 3 Experimental Design, Materials and Methods

The database includes 29,495 photographic portraits collected on 397 individuals from the only wild social group of mandrills habituated to human presence. This group, which roams in the Lékédi Park and its surroundings, in southern Gabon (near to the village of Bakoumba), is daily monitored by the Mandrillus Project [1] (www.projetmandrillus.com) for researches in ecology and evolution (see for examples: [2,3]). The group was founded after the release of 65 semi-captive individuals (born and raised at CIRMF; Centre International de Recherches Médicales de Franceville, Gabon), in 2002 (36 individuals) and 2006 (29 individuals [4]). Starting as early as 2003, wild males joined the group to reproduce with released females. In 2021, most of the individuals of the group (>95%) were wild-born. Photos were taken directly in the forest by field assistants while following the study group. Since the beginning of the project, field assistants have used different models of DSLR cameras and long-focal lenses (varying from 70 to 500 mm, depending on camera models and distance to the subjects). Photos are uploaded on a computer regularly, and renamed by the assistants using the syntax presented above. Co-authors of this article (BRT, MH, MJEC, LS), who know the identity of the studied mandrills, monthly validated the names of the individuals depicted on all pictures from the database. Pictures were then processed using Adobe Photoshop Lightroom Software version 10.1.1. Images were first oriented to align the pupils of the eyes horizontally, and then centered and cropped to keep only the face (removing the neck and the ears). No further processing was applied.

## Ethics Statements

The Mandrillus Face Database is based on non-invasive methods (pictures taken during the daily routine of the animals). Pictures were taken from a distance and without a flashlight. Photographers took pictures on the fly without any obvious perturbation of the study mandrills. These mandrills have been habituated to human presence since 2012. The Mandrillus Project and associated studies have been approved by an authorization from the CENAREST institute (permit number, AR017/22/MESRSTTCA/CENAREST/CG/CST/CSAR).

## CRediT Author Statement

**Sonia Tieo:** Data curation, Writing - Original draft. **Claudia Ximena Restrepo-Ortiz:** Data curation. **Marie Charpentier:** Data curation, Supervision, Reviewing and Editing. **Julien P. Renoult:** Data curation, Supervision, Reviewing and Editing. **Berta Roura-Torres:** Data curation, Photography. **Mélanie Harté:** Data Curation. **Loic Sauvadet:** Photography.

## Declaration of Competing Interest

The authors declare that they have no known competing financial interests or personal relationships that could have appeared to influence the work reported in this paper.

The authors declare the following financial interests/personal relationships which may be considered as potential competing interests:

## Data Availability

Mandrillus Face Database (Original data) (Zenodo).
